# TLM-Quant: An Open-Source Pipeline for Visualization and Quantification of Gene Expression Heterogeneity in Growing Microbial Cells

**DOI:** 10.1371/journal.pone.0068696

**Published:** 2013-07-17

**Authors:** Sjouke Piersma, Emma L. Denham, Samuel Drulhe, Rudi H. J. Tonk, Benno Schwikowski, Jan Maarten van Dijl

**Affiliations:** 1 Department of Medical Microbiology, University of Groningen and University Medical Center Groningen, Groningen, The Netherlands; 2 Institut Pasteur, Systems Biology Lab, Department of Genomes and Genetics, Paris, France; University of Connecticut, United States of America

## Abstract

Gene expression heterogeneity is a key driver for microbial adaptation to fluctuating environmental conditions, cell differentiation and the evolution of species. This phenomenon has therefore enormous implications, not only for life in general, but also for biotechnological applications where unwanted subpopulations of non-producing cells can emerge in large-scale fermentations. Only time-lapse fluorescence microscopy allows real-time measurements of gene expression heterogeneity. A major limitation in the analysis of time-lapse microscopy data is the lack of fast, cost-effective, open, simple and adaptable protocols. Here we describe TLM-Quant, a semi-automatic pipeline for the analysis of time-lapse fluorescence microscopy data that enables the user to visualize and quantify gene expression heterogeneity. Importantly, our pipeline builds on the open-source packages ImageJ and R. To validate TLM-Quant, we selected three possible scenarios, namely homogeneous expression, highly ‘noisy’ heterogeneous expression, and bistable heterogeneous expression in the Gram-positive bacterium *Bacillus subtilis.* This bacterium is both a paradigm for systems-level studies on gene expression and a highly appreciated biotechnological ‘cell factory’. We conclude that the temporal resolution of such analyses with TLM-Quant is only limited by the numbers of recorded images.

## Introduction

Microorganisms need to adapt to environmental changes by appropriately adjusting their gene expression [1]. They can achieve this through carefully controlled signal transduction pathways that modulate the transcription of individual genes. In recent years it has become increasingly clear that the expression of particular genes is often not uniform in the individual cells of a microbial population, even when these cells are grown under carefully controlled conditions. Firstly, there can be considerable noise or heterogeneity in the expression levels of individual genes, and secondly, there can even be situations of bistability where particular genes are only transcribed in a sub-population of the analysed cells. A paradigm for studies on gene expression heterogeneity is the bacterium *Bacillus subtilis*. Individual *B. subtilis* cells within a population can, for example, differentiate into a motile state for migration to more favourable environments, a competent state to take up DNA from the environment, or a dormant state in the form of spores [2], 3. Microbial gene expression heterogeneity also has important biotechnological implications since, for obtaining the highest product yields, all microbes used in industrial-scale fermentations should express the gene(s) of interest at the highest possible level; poorly producing cells are unwanted [4].

The theoretical and practical ramifications of gene expression heterogeneity have led to a strong interest in effective tools to monitor and quantify this phenomenon. Most strategies involve the fusion of the promoter sequence of a gene of interest to a promoter-less copy of the gene encoding the Green Fluorescent Protein (GFP). Overall promoter activity and expression of the gene of interest can then be determined by fluorescence readings of culture samples. This is achieved in real time using suitable plate reader assays [5]–[7]. To investigate gene expression heterogeneity in different cells of growing populations, alternative approaches are needed, such as flow cytometry and time-lapse microscopy. Only time-lapse microscopy allows real-time measurements, and this technique is substantially less laborious than flow cytometry. Different time-lapse microscopy set-ups have been described in the recent literature [8]–[10]. Though very effective, a significant drawback of these approaches is that the downstream data analysis usually requires expensive, highly sophisticated, and/or custom-made software [9]–[12]. Since we needed a simple and readily adaptable tool for the quantitative analysis of large amounts of time-lapse microscopy data, we established the TLM-Quant pipeline for data processing and analyses based on open-source software. This pipeline was validated using a custom-built fluorescence microscopy set-up and *B. subtilis* strains producing GFP from promoters that direct either homogenous, heterogeneous, or bistable gene expression, as described by Botella *et al.* [6]. Importantly, the TLM-Quant pipeline was then effectively implemented in a large-scale systems biological analysis on the global network reorganization during dynamic adaptations of *B. subtilis* metabolism to nutritional shifts between the preferred carbon sources glucose and malate [7]. In the latter study, TLM-Quant allowed us to verify the absence of heterogeneity in the expression of genes involved in central carbon metabolism. The respective datasets can be queried at https://basysbio.ethz.ch/openbis/index.html?viewMode=SIMPLE#action=DOWNLOAD_ATTACHMENT&file=populationhomogeneity.pdf&&entity=PROJECT&code=BASYSBIO_BIG&space=BASYSBIO_PUBLIC or http://tinyurl.com/basysbiodata. A detailed description of TLM-Quant as presented here and in the Tutorial S1 was however not published thus far.

## Analysis

**Figure 1:**
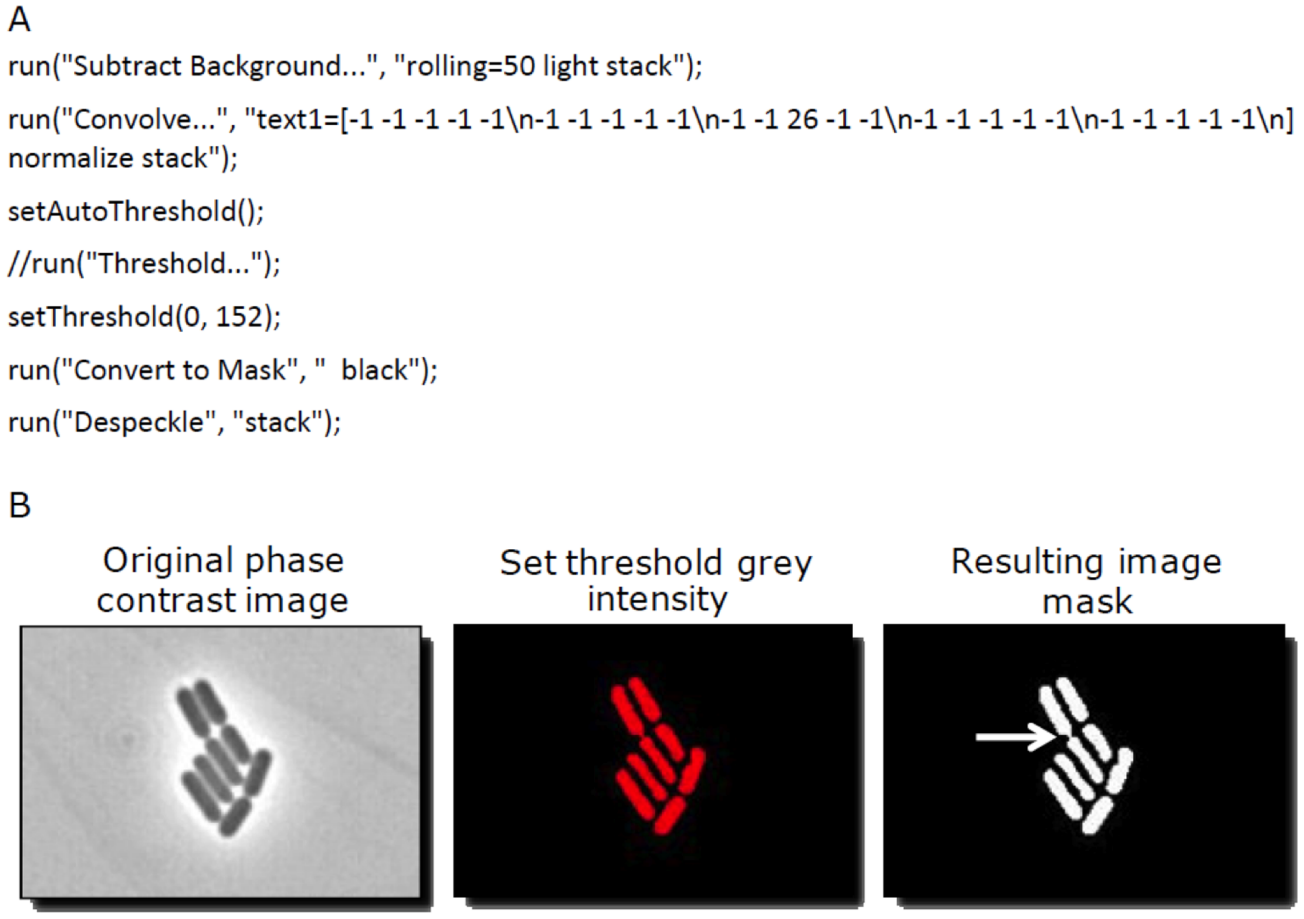
Processing phase contrast images to create segmented cells. (A) ImageJ commands for the processing of phase contrast images to create segmented cells. (B) Visualization of the image processing from the original phase contrast image, through background subtraction, convolution, setting of a threshold grey intensity, conversion of values within threshold to mask and de-speckling. Red objects in the processed image are above the threshold and counted as cells. Notably, non-separated pairs of cells as marked with the white arrow pointing at the site of their attachment will be counted as one cell.

For image analysis by TLM-Quant, we will assume that, for each time point, a phase-contrast image and an overlapping fluorescent image are available, both encoded in 8-bits (intensity from 0 to 255). Downstream processing can be generalized to multiple channels (colours). To visualise and quantify gene expression heterogeneity, the fluorescence information in the recorded images is extracted using ImageJ software (available via http://rsbweb.nih.gov/ij/) [12]. To obtain correct cellular fluorescence measurements, cells are segmented in phase contrast images by using the commands ‘Subtract background’ and ‘Convolve’. The kernel used in the ‘Convolve’ command is specified in Figure 1A and should be adjusted depending on cell type and exposure time. A copy of the obtained image is converted to a binary mask (intensity 0 or 255) using the ‘apply’ command in the threshold dialogue. Figure 1 shows the ImageJ macro commands for this process and illustrates its performance starting from an original phase contrast image. The pixel intensities from the fluorescence image are then subtracted from the mask. This yields cells with inverted intensities that are analysed by setting a threshold for all grey values but the minimal grey value, and by subsequently executing the ‘analyse particles’ command. The original intensities are then recovered by subtracting the negative intensities from 255. To measure background fluorescence, the fluorescence images are first added to the mask, and only values below 255 are collected. In this way the entire area in the image, except the cell areas, is analysed using the ImageJ ‘analyse particles’ command and the returned value represents the average background fluorescence.

For statistical analyses and data processing in R [13], [14], [15], the data from ImageJ are saved in CSV format. The derived normalised fluorescence intensities are obtained using the formula:

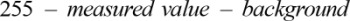



## Results and Discussion

**Figure 2:**
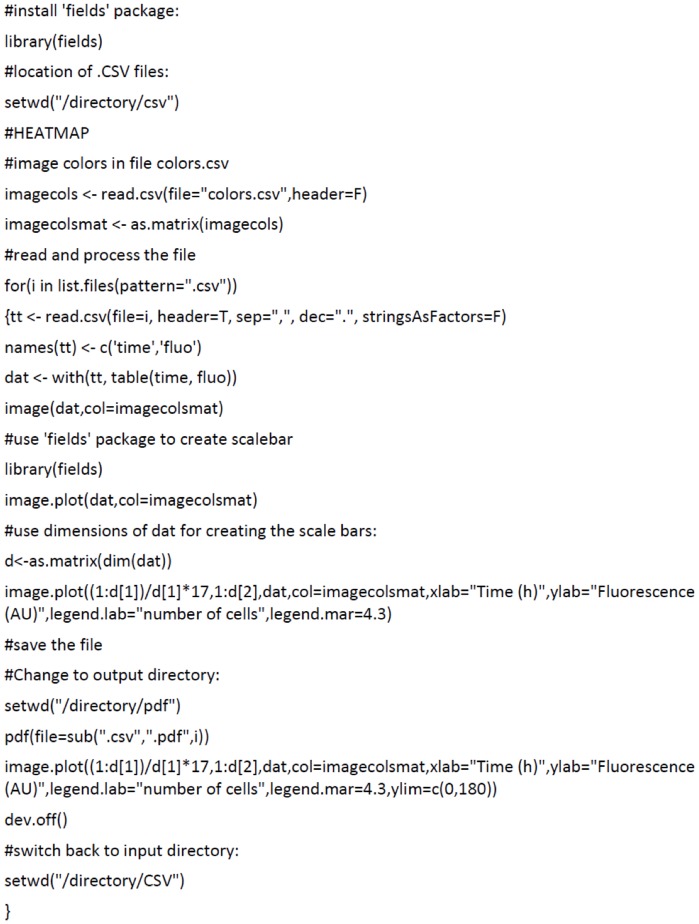
Script used in the R software package to generate heatmap plots from ImageJ output data. Note that the indicated directories are arbitrary examples. The colors.csv file used in this script can be altered for implementing other color schemes.

**Figure 3:**
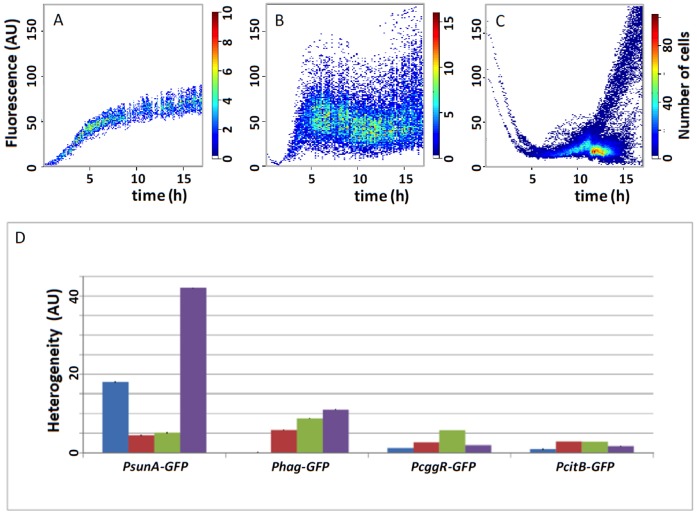
Heatmap plots created using the R script in Figure 2. (**A**) *B. subtilis* cells expressing a fusion of the IPTG-inducible P*spac* promoter with GFP [6], [7] show homogeneous fluorescence. (**B**) *B. subtilis* cells expressing a fusion of the authentic promoter of the *sunA* gene to GFP show heterogeneous fluorescence when grown on a Luria Bertani agarose medium. (**C**) *B. subtilis* cells expressing the same *sunA* promoter GFP fusion as in B show bistable heterogeneous fluorescence when grown on an M9 agarose medium. Note that at early time points already two populations of cells with differing fluorescence intensities can be distinguished. AU, arbitrary units. (**D**) Bar diagrams for easy comparison of the outcomes of multiple heterogeneity measurements during growth on M9 medium as shown in panels A-C. At t = 2 h, cells are in the exponential growth phase (blue bars); at t = 5 h, the highest numbers of cells are observed (red bars); at t = 10 h, a minimum in the cell numbers has been reached due to cell death (green bars); and at t = 17 h, the surviving cells have resumed growth (purple bars). Heterogeneity is expressed in arbitrary units (AU).

The code in Figure 2 is used to create plots as shown in Figure 3 (A-C). Importantly, the R script allows processing of data from many experiments in a short time period. The output is a high-resolution PDF file visualizing the levels of expression heterogeneity. Figure 3 illustrates three possible heterogeneity scenarios, namely homogeneous expression (A), ‘highly noisy’ heterogeneous expression, (B) and bistable heterogeneous expression (C).

**Figure 4:**
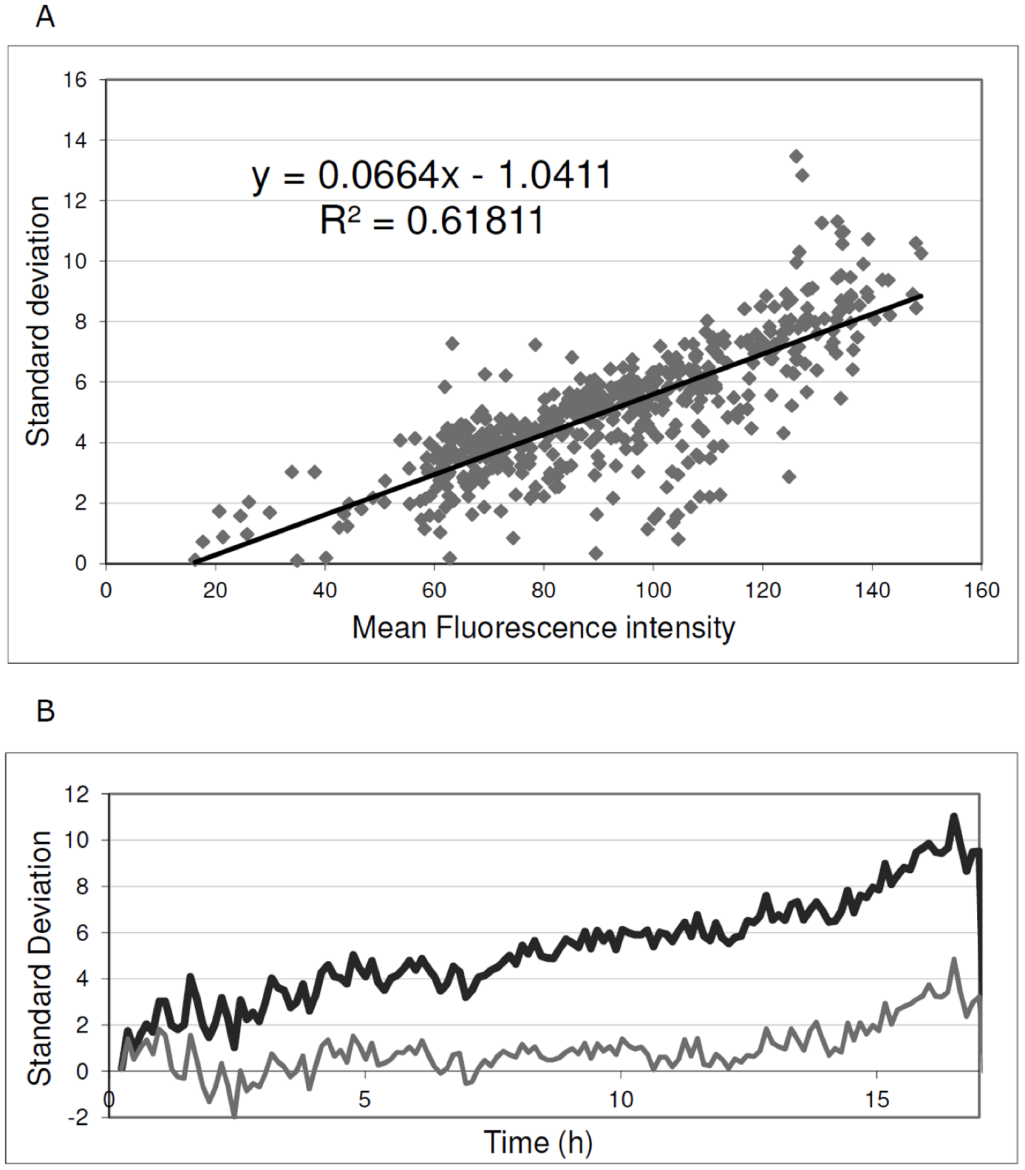
Quantification of expression heterogeneity. To obtain baseline values for GFP expression heterogeneity, a *B. subtilis* P*spac*-GFP strain was used in which the fluorescence intensity of the cells can be varied by varying the amount of the inducer IPTG in the growth medium. Importantly, the variation in GFP fluorescence in this cell population is minimal compared to cells expressing GFP from non-engineered promoters. Therefore, the observed variation can be regarded as a baseline for GFP expression heterogeneity. (**A**) Standard deviation in the fluorescence intensity of individual cells of *B. subtilis* P*spac*-GFP as a function of the mean fluorescence intensity of the cell population. The analysis included 535 measurements collected from four cultures supplemented with IPTG to 0.05 mM, 0.1 mM, 0.5 mM, or 1 mM. (**B**) Example to illustrate the effectiveness of the applied heterogeneity correction. When applied to a P*spac*-GFP strain grown in the presence of 0.05 mM IPTG the correction shows close to zero levels of expression heterogeneity over a period of at least 12 h. At later time points cells started to lyse in this experiment resulting in a slightly increased GFP expression heterogeneity. Black line, raw fluorescence data; Grey line, P*spac*-GFP subtracted data.

We recommend inclusion of controls for homogenous expression, as in Figure 3A, in all analyses to measure background levels of expression noise. For studies in *B. subtilis*, we have implemented an *amyE*::P*spac*-GFPmut2 strain in which homogeneous GFP expression can be set at different levels by growing the cells in the presence of different IPTG concentrations. This is due to the fact that, in this particular strain, the transcription of *gfp* is driven by the IPTG-dependent P*spac* promoter. Specifically, we added IPTG to the growth medium at concentrations of 0.05 mM, 0.1 mM, 0.5 mM, or 1 mM and performed time-lapse fluorescence microscopy. As expected, this resulted in homogenous expression of GFP in exponentially growing cells, but at different levels depending on the IPTG concentration in the growth medium. Next, the standard deviation in cellular fluorescence in one time-lapse image was plotted as a function of the mean cellular fluorescence intensity in that particular time-lapse image (Figure 4A). Importantly, the standard deviation in the cellular fluorescence, which essentially represents the background noise when GFP is homogeneously expressed, showed a linear correlation with the mean fluorescence intensity. Accordingly, the corresponding regression line equation can be used for background noise correction in other analyses. This is illustrated in Figure 4B, where the correction is applied to the P*spac*-GFP strain grown in the presence of 0.05 mM IPTG.

As exemplified in Figure 3D, large numbers of heterogeneous gene expression measurements at different time points and for different promoter-GFP fusions can be readily compared using bar charts generated in spread sheet editors, like LibreOffice Calc or Microsoft Excel [7]. In this case, averages and standard deviations are calculated from the combined fluorescence values. However, the bar charts do not discriminate between highly heterogeneous and bistable gene expression. Notably, Microsoft Excel is not ‘open source’, but since the vast majority of potential users of TLM-Quant have easy access to Excel, we should mention this option.

In conclusion, time-lapse microscopy is currently the only method that allows real-time measurements of promoter activity, reflected by GFP expression, at the single cell level. Here we document the TLM-Quant pipeline, which allows the user to readily visualise and quantify gene expression heterogeneity using freely available open-source tools. Importantly, this pipeline is simple and robust – there are almost no thresholds and parameters to fine-tune. Since TLM-Quant is based on free open-source tools that almost every one can master, it is easy to adapt to a wide range of different – and potentially new – types of images. Thus, while we describe the use of TLM-Quant for the soil bacterium *B. subtilis* 168, the established scripts can be applied to studies on gene expression heterogeneity in all other microorganisms that can be grown in a time-lapse microscopy system.

## Supporting Information

Tutorial S1 **The Tutorial includes detailed instructions for the implementation of TLM-Quant**.
(PDF)
