# Supplementary material for: TLM-Quant: An Open-Source Pipeline for Visualization and Quantification of Gene Expression Heterogeneity in Growing Microbial Cells
Source: PLoS One. 2013 Jul 17;8(7):e68696. doi: 10.1371/journal.pone.0068696 (PMC3714294; doi:10.1371/journal.pone.0068696)
Supplement: Tutorial S1 — The Tutorial includes detailed instructions for the implementation of TLM-Quant. (PDF) [file pone.0068696.s001.pdf]

Supplementary Tutorial S1:

Standard Operating Procedure (SOP) TLM-Quant Image Analysis

Description

The TLM-Quant image analysis pipeline is intended for analysis of the expression of promoter-GFP fusions in growing cells using time-lapse microscopy. The specific parameters have been set for measurements of GFP expression in *B. subtilis*, but the user can adjust the parameters to fit specific experimental requirements. Images are analyzed in ImageJ and the raw data is further processed in LibreOffice or Excel and R. The output consists of growth curves, fluorescence intensity per cell and heterogeneity in the population. The menu path to a command in ImageJ is written in italics (e.g.: *Edit/Options/Memory & Theads*).

Materials

Hardware

Your computer should have 2GB of RAM memory for ImageJ to function properly.

Software

- MacBiophotonics ImageJ (http://www.macbiophotonics.ca/imagej/)
  - Set memory usage of ImageJ to 1500MB: *Edit/Options/Memory & Theads*
- Microsoft Excel
- R

Method

Macbiophotonics ImageJ

- Open the phase contrast images
  - *File/import/image sequence*
  - Double-click on a random image in the right folder
  - Select the correct images by typing in the box “file name contains”
- Run the macro *phase contrast processing* (Appendix A)
  All cells will now have maximum intensity
- Open the fluorescence images
  - *File/import/image sequence*
  - Double-click on a random image in the right folder
  - Select the correct images by typing in the box “file name contains”
- Subtract fluorescent image from phase contrast image
  - *Process/Image Calculator*
  - Operation: subtract

  - Image1 is phase contrast, Image2 is Fluorescence image

  Cells will now have an inverted fluorescence intensity. This is reverted in Excel by subtracting this value from the maximum intensity
- Set threshold
  - *Image/adjust/threshold*
  - Set the threshold 1 to 255
- Analyze the cells:
  - *Analyze/Set Measurements*
  - Measure: Area, Standard deviation, Min&max Gray values, Mean Gray value, Feret's Diameter, Slice number
  - *Analyze/Analyze Particles*
    - Size: 10-infinity (adjust according to cell size of interest)
    - Circularity: 0.00-1.00
    - Show: Nothing
    - Display results
    - Clear results
    - Summarize
    - Include holes
  - In the results window:
    - Go to last result and click on it
    - Scroll all the way up using the scrollbar
    - Press and hold shift while clicking the first result (effectively making a selection of the whole results table)
    - Press ctrl+c
    - Paste in the yellow cell in the – Template Microscopy – excel sheet

ImageJ (background subtraction)

We use a second ImageJ version to avail of two different sets of measurements in the *analyze particles* command, so that there is no need to change them each time.

- Open the fluorescence images
  - *File/import/image sequence*
  - Double-click on a random image in the right folder
  - Select the correct images by typing in the box "file name contains" (e.g. fl_1)
- Make a selection of an area where no cells are present throughout the stack
  - Hold the left mouse button while drawing a selection
  - If necessary, temporarily enhance the contrast (**do not click apply**) to locate
    - *Image/adjust/brightness&contrast*
- Set threshold
  - *Image/adjust/threshold*
  - Set the threshold between 0 and 254

- Analyze the background:
    - *Analyze/Set Measurements*
    - Measure: Mean Gray value
    - *Analyze/Analyze Particles*
        - Size: 0-infinity
        - Circularity: 0.00-1.00
        - Show: Nothing
        - Display results
        - Clear results
        - Summarize
    - In the results window:
        - **Check that the number of results is equal to the number of images in the stack**
        - Go to last result and click on it
        - Scroll all the way up using the scrollbar
        - Press and hold shift while clicking the first result (effectively making a selection of the whole results table)
        - Press ctrl+c
        - Paste in the yellow cell in the – Template Microscopy – Excel sheet

- Generate heatmap plots from the Excel sheet using the script for the R software package as presented in Appendix B.
    - Paste time points (column A) and mean fluorescence (column B) values in Excel sheet (without headers)
    - Save the resulting Excel sheet as .csv file
    - Save Appendix C as a .csv file in a separate directory
    - Define directories to your preference by adjusting them at the command setwd (4 locations, high-lighted in yellow)
    - Run R script from Appendix B.

Appendix A: ImageJ Macro

Phase contrast processing:

```
run("Subtract Background...", "rolling=50 light stack");
run("Convolve...", "text1=[-1 -1 -1 -1 -1\n-1 -1 -1 -1 -1\n-1 -1 26 -1 -1\n-1 -1 -1 -1 -1\n-1 -1 -1 -1 -1\n] normalize stack");
setAutoThreshold();
//run("Threshold...");
setThreshold(0, 152);
run("Convert to Mask", "  black");
run("Despeckle", "stack");
```

Appendix B: Script uin the R software package to generate heatmap plots from ImageJ output data

```
#install 'fields' package:
library(fields)
#location of colors.csv file:
setwd("/directory/csv")
#HEATMAP
#image colors in file colors.csv
imagecols <- read.csv(file="colors.csv",header=F)
imagecolsmat <- as.matrix(imagecols)
#read and process the file
#location of .CSV files:
setwd("/directory/csv")
for(i in list.files(pattern=".csv"))
{tt <- read.csv(file=i, header=T, sep=",", dec=".", stringsAsFactors=F)
names(tt) <- c('time','fluo')
dat <- with(tt, table(time, fluo))
image(dat,col=imagecolsmat)
#use 'fields' package to create scalebar
library(fields)
image.plot(dat,col=imagecolsmat)
#use dimensions of dat for creating the scale bars:
d<-as.matrix(dim(dat))
image.plot((1:d[1])/d[1]*17,1:d[2],dat,col=imagecolsmat,xlab="Time
(h)",ylab="Fluorescence (AU)",legend.lab="number of
cells",legend.mar=4.3)
#save the file
#Change to output directory:
setwd("/directory/pdf")
pdf(file=sub(".csv",".pdf",i))
image.plot((1:d[1])/d[1]*17,1:d[2],dat,col=imagecolsmat,xlab="Time
(h)",ylab="Fluorescence (AU)",legend.lab="number of
cells",legend.mar=4.3,ylim=c(0,180))
dev.off()
#switch back to input directory:
setwd("/directory/CSV")
}
```

Appendix C: Heatmap colors

#FFFFFF
#000093
#000097
#00009B
#00009F
#0000A3
#0000A7
#0000AB
#0000AF
#0000B3
#0000B7
#0000BB
#0000BF
#0000C3
#0000C7
#0000CB
#0000CE
#0000D3
#0000D7
#0000DA
#0000DE
#0000E2
#0000E6
#0000EA
#0000EE
#0000F3
#0000F7
#0000FB
#0000FF
#0002FF
#0006FF
#000AFF
#000FFF
#0013FF
#0017FF
#001BFF
#001FFF
#0023FF
#0027FF
#002BFF
#002FFF
#0033FF
#0037FF
#003BFF
#003FFF
#0043FF
#0047FF
#004BFF
#004EFF
#0052FF
#0056FF
#005AFF
#005EFF
#0062FF

#0066FF
#006AFF
#006EFF
#0072FF
#0076FF
#007AFF
#007EFF
#0082FF
#0086FF
#0089FF
#008DFF
#0091FF
#0095FF
#0099FF
#009DFF
#00A1FF
#00A5FF
#00A9FF
#00ADFF
#00B1FF
#00B5FF
#00B9FF
#00BDFF
#00C1FF
#00C5FF
#00C8FF
#00CCFF
#00D0FF
#00D4FF
#00D8FF
#00DCFF
#00E0FF
#00E4FF
#00E8FF
#00ECFF
#00F0FF
#00F5FF
#00F9FF
#00FDFF
#01FFFE
#04FFFB
#08FFF7
#0CFFF3
#11FFEE
#15FFEA
#19FFE6
#1DFFE2
#21FFDE
#25FFDA
#29FFD6
#2DFFD2
#31FFCE
#35FFCA
#39FFC6
#3DFFC2
#41FFBE
#45FFBA

#48FFB6
#4CFFB3
#50FFAF
#54FFAB
#58FFA7
#5CFFA3
#60FF9F
#64FF9B
#68FF97
#6CFF93
#70FF8F
#74FF8B
#78FF87
#7CFF84
#80FF80
#84FF7C
#87FF78
#8BFF74
#8FFF70
#93FF6C
#97FF68
#9BFF64
#9FFF60
#A3FF5C
#A7FF58
#ABFF54
#AFFF50
#B3FF4C
#B7FF48
#BBFF44
#BFFF40
#C3FF3C
#C6FF39
#CAFF35
#CEFF31
#D2FF2D
#D6FF29
#DAFF25
#DEFF21
#E2FF1D
#E6FF19
#EAFF15
#EEFF11
#F3FF0C
#F7FF08
#FBFF04
#FEFF01
#FFFD00
#FFF900
#FFF500
#FFF000
#FFEC00
#FFE800
#FFE400
#FFE000
#FFDC00
#FFD800

#FFD400
#FFD000
#FFCC00
#FFC800
#FFC400
#FFC000
#FFBC00
#FFB900
#FFB500
#FFB100
#FFAD00
#FFA900
#FFA500
#FFA100
#FF9D00
#FF9900
#FF9500
#FF9100
#FF8D00
#FF8900
#FF8600
#FF8200
#FF7E00
#FF7A00
#FF7600
#FF7200
#FF6E00
#FF6A00
#FF6600
#FF6200
#FF5E00
#FF5A00
#FF5600
#FF5200
#FF4E00
#FF4A00
#FF4600
#FF4200
#FF3E00
#FF3B00
#FF3700
#FF3300
#FF2F00
#FF2B00
#FF2700
#FF2300
#FF1F00
#FF1B00
#FF1700
#FF1300
#FF0F00
#FF0A00
#FF0600
#FF0200
#FE0000
#FB0000
#F70000

#F30000
#EE0000
#EA0000
#E60000
#E20000
#DE0000
#DA0000
#D60000
#D20000
#CE0000
#CA0000
#C60000
#C20000
#BE0000
#BB0000
#B70000
#B30000
#AF0000
#AB0000
#A70000
#A30000
#9F0000
#9B0000
#970000
#930000
#8F0000
#8B0000
#870000
#840000
#800000
